# Does Pentaerytrithyltetranitrate reduce fetal growth restriction in pregnancies complicated by uterine mal-perfusion? Study protocol of the PETN-study: a randomized controlled multicenter-trial

**DOI:** 10.1186/s12884-019-2456-7

**Published:** 2019-09-14

**Authors:** T. Groten, T. Lehmann, E. Schleußner, for the PETN Study Group, Ulrich Pecks, Constantin von Kaisenberg, Mateja Condic, Stefan Verlohren, Dietmar Schlembach, Clinic Neukoelln, Gregor Seliger, Sven Seeger, Anne Tauscher, Matej Komar, Karl Oliver Kagan, Ulrike Friebe-Hoffmann, Christoph Hübener, Laura de Vries

**Affiliations:** 1 Department of Obstetrics, University Hospital Jena, Am Klinkum 1, 07740 Jena, Germany; 2 Institute of Medical Statistics and Computer Science, University Hospital Jena, Friedrich Schiller University Jena, Jena, Germany

**Keywords:** Fetal growth restriction, Abnormal uterine Doppler, Nitric oxide (NO-) donors, Pentaerytrithyltetranitrate (PETN), Perinatal death

## Abstract

**Background:**

Affecting approximately 10% of pregnancies, fetal growth restriction (FGR), is the most important cause of perinatal mortality and morbidity. Impaired placental function and consequent mal-perfusion of the placenta is the leading cause of FGR. Although, screening for placental insufficiency based on uterine artery Doppler measurement is well established, there is no treatment option for pregnancies threatened by FGR. The organic nitrate pentaerithrityl tetranitrate (PETN) is widely used for the treatment of cardiovascular disease and has been shown to have protective effects on human endothelial cells. In a randomized placebo controlled pilot-study our group could demonstrate a risk reduction of 39% for the development of FGR, and FGR or death, by administering PETN to patients with impaired uterine artery Doppler at mid gestation. To confirm these results a prospective randomized placebo controlled double-blinded multicentre trial was now initiated.

**Method:**

The trial has been initiated in 14 centres in Germany. Inclusion criteria are abnormal uterine artery Doppler, defined by mean PI > 1.6, at 19^0^ to 22^6^ weeks of gestation in singleton pregnancies. Included patients will be monitored in 4-week intervals. Primary outcome measures are development of FGR (birth weight < 10th percentile), severe FGR (birth weight < 3rd centile) and perinatal death. Placental abruption, birth weight below the 3rd, 5th and 10th centile, development of FGR requiring delivery before 34 weeks` gestation, neonatal intensive care unit admission, and spontaneous preterm delivery < 34 weeks` and 37 weeks` gestation will be assessed as secondary endpoints. Patient enrolment was started in August 2017. Results are expected in 2020.

**Discussion:**

During the past decade therapeutic agents with possible perfusion optimizing potential have been evaluated in clinical trials to treat FGR. Meta-analysis and sub-analysis of trials targeting preeclampsia revealed ASS to have a potential in reducing FGR. Phosphodiesterase-type-5 inhibitors have recently been tested in a worldwide RCT for therapy of established FGR, failing to show an effect on neonatal outcome. The ongoing multicenter trial will, by confirming our previous results, finally provide a therapeutic option in cases at risk for FGR.

**Trial registration:**

DRKS00011374 registered at September 29th, 2017 and NCT03669185, registered September 13th, 2018.

## Background

Affecting approximately 10% of pregnancies, fetal growth restriction (FGR), is the most important cause of perinatal mortality and morbidity. Mortality rates are increased in FGR at any gestational age, and in combination with preterm delivery FGR is associated with higher rates of cerebral palsy, sensory deficits, learning disabilities, and respiratory illnesses [1]. Additionally, individuals born growth restricted are facing lifelong consequences as FGR accounts as developmental origin of adult diseases [2]. The leading cause of FGR is mal-perfusion of the placenta based on insufficient transformation of the uterine arteries and impaired placental function [3]. Uterine artery Doppler measurement is used to determine utero-placental perfusion during pregnancy and thus is established as Screening for placental insufficiency [4, 5].

So far there is no treatment option for pregnancies complicated by FGR and the clinical management is restricted to close monitoring and assessing for the optimal time point of delivery; pondering between intrauterine hypoxia and extrauterine prematurity. Enormous studies have been undertaken to reveal the usefulness of low-dose aspirin (LDA) or antioxidants for secondary and primary prevention of FGR [6, 7]. Although LDA is effective for primary prevention, if initiated before 16 weeks gestation [8, 9], there is still no treatment option for those recognized beyond 20 weeks.

The organic nitrate pentaerithrityl tetranitrate (PETN) is commonly used in the treatment of angina pectoris. As a No-donor it improves perfusion and oxygen supply to the myocardium. In disparity to other NO-donors, aside from vasodilation, PETN also possesses potent endothelium protective qualities by enhancing the expression of antioxidant genes, like heme oxygenase-1 (HO-1), in human endothelial cells [10]. The described biological effects and its well-documented biological safety unveiled PETN to possibly be effective and safe for secondary prevention in pregnancies complicated by and at risk of FGR.

The clinical benefit of the prophylactic use of a NO-donor in pregnant women at risk was first demonstrated by Lees et al. [11]. In this randomized, placebo-controlled, blinded trial glycerol trinitrate patches significantly increase the likelihood of a complication-free pregnancy (*p* = 0.004) and reduced hazard by 73%. Our group could demonstrate a risk reduction of 39% (relative risk RR = 0.609, 95% CI 0.367 to 1.011) for the development of FGR and for FGR combined with perinatal death (RR = 0.615, 95% CI 0.378 to 1.000) through administration of PETN to patients recognized by impaired uterine artery Doppler at mid gestation in a randomized placebo controlled pilot study [12]. Furthermore, a risk reduction of 49% for FGR, as well as for FGR combined with perinatal death (RR = 0.509 (95% CI 0.257 to 1.007), could be achieved in those patients being at high risk, recognized by impaired placental perfusion and an additional history of former pregnancy complications, in the PETN group [12].

These results reveal for the first time, that PETN indeed offers a clinically relevant option to improve fetal outcome. However, the number of 111 enrolled patients does not create enough power to ultimately prove the beneficial effect. Therefore, it is especially important to confirm these results and to verify the efficacy and safety of PETN in a randomized blinded placebo-controlled multicentre-trial with an appropriately calculated sample size. The study protocol of the PETN Trial will be described in detail below.

## Methods/design

### Study objective & design

The aim of this study is to confirm the safety and effectiveness of PETN for secondary prophylaxis of FGR in a prospective, multicenter, randomized, double-blind, placebo controlled, parallel group, interventional trial.

### Ethics statement

Ethical approval has been obtained from the ethical committee of the University Hospital of Jena as leading committee (Institutinal review board (IRB) Friedrich Schiller University, Jena) (5085–02/17). Each of the 14 German study centers obtained ethical approval of their local committees (IRB Universitäts-Kinderklinik, Kiel, IRB Hannover Medical School, Hannover, IRB Medical School, Rheinischen Friedrich-Wilhelms-University, Bonn, IRB State Office for Health and Social Affairs (LAGeSo) Berlin (two centres), IRB of the state of Saxony-Anhalt, Halle (two centres), IRB Medical School, Leipzig, IRB Technical University Dresden, IRB Medical School, Eberhard-Karls-University, Tübingen, IRB University of Ulm, IRB Ludwig Maximilian University, Munich, IRB national Medical association Bavaria, Munich), which than were referred to the Jena committee before approval was issued. Written informed consent must be obtained before the performance of any protocol related procedures that are not part of usual subject care.

### Inclusion criteria

Eligible patients for the proposed trial are pregnant women recognized to be at risk of adverse pregnancy outcome associated with FGR at mid gestation. Since poor placental function and mal-perfusion of the placenta is recognized to cause FGR [3] impaired placental flow measurement is strongly associated with the development of FGR. A mean pulsatility index >95th percentile according to Cnossen et al. predicts FGR with a sensitivity of 18% with a specificity of 95% in a general population and a sensitivity of 58% for any FGR in a high risk population with a specificity of 75% [4]. The risk to develop FGR is in general higher in women with a medical history of chronic hypertension, diabetes mellitus type 2, systemic lupus erythematodes or antiphopholipid antibody syndrome and in those with prior history of adverse pregnancy outcome associated with FGR (late pregnancy loss, stillbirth, abruption placentae, preterm delivery, preeclampsia or HELLP-syndrome) [13]. Accordingly, at least one third of the pregnant women diagnosed with impaired perfusion of the uterine arteries at mid gestation will develop FGR and associated adverse pregnancy outcomes. In the presence of additional risk factors this likelihood will rise considerably.

Consequently, the Inclusion criteria are impaired uterine artery Doppler ultrasound, defined by a mean Pulsatility Index (PI) greater than 1.6 (> 95th centile of German reference population, PIA Fetal Database, Version 4.00 and higher, Viewpoint, Germany; [14]), singleton pregnancy at 19^0^ to 22^6^ weeks of gestation who are over 18 years old and provide informed consent.

### Exclusion criteria

To ensure the inclusion of solely placental reasons for FGR and for ethical reasons, exclusion criteria are multiple pregnancies, fetal chromosomal or suspected major structural defects, circumstances predicting adverse pregnancy outcome other than due to FGR, like premature rupture of membrane or cervical insufficiency at the time of enrolment, participation in any other clinical trial and maternal disease defined as contraindication for intake of PETN.

### Primary outcome measures

As primary efficacy endpoint development of FGR (< birth weight below the 10th centile) or perinatal death as a composite outcome was proposed in accordance to the pilot study [12]. Key secondary endpoints are severe morbidity and mortality as composite of severe FGR, intrauterine or neonatal death or placental abruption, birth weight below the 3rd, 5th and 10th centile according to Voigt [15], development of FGR requiring delivery before 30 and before 34 weeks` gestation, spontaneous preterm delivery before 34 weeks and before 37 weeks gestation, rate of neonatal intensive care unit admission and neonatal complications like the need of ventilation, occurrence of intraventricular hemorrhage II – IV ° or necrotized enterocolitis.

### Determination of primary and secondary measures

The gestational age was calculated from a certain last menstrual period date and confirmed by first trimester measurement of the crown-rump-length. Weight percentiles are derived from gestational age and weight according to the German standards by Voigt [15]. Falling below the 10th weight percentile in combination with impaired uterine perfusion (inclusion criteria mean PI of > 1.6) is defined as FGR [16].

Development of severe FGR is defined as birth weight below the 5th centile. Preterm delivery is determined from gestational age at birth.

### Methods against bias

Patients will be randomly assigned to treatment groups in blocks of varying size. Randomization will be stratified by center and by an indicator for low or high risk of FGR. Patients will be classified as high risk in case of pre-existing hypertension, pre-existing diabetes or obstetric history of FGR, late pregnancy loss, stillbirth, abruption placentae, preterm delivery, preeclampsia or HELLP-syndrome. All others are at low risk. PETN and placebo will be administered in a double-blind fashion. Primary analysis will be performed in the intent-to-treat population.

### Proposed sample size/power calculations

Sample size calculation is based on results generated from our pilot trial [12]. Since additional risk factors increase the risk of FGR a stratified randomisation and analysis is planned for two strata. A 2:1 ratio was observed for low and high risk patients in the trial. Proportion of FGR or perinatal death was 0.42 in the low-risk and 0.69 in the high-risk stratum of the placebo group. We discovered an odds ratio of 0.41 (PETN vs. placebo) in the pilot trial. More conservatively, we want to be able to find a 50% risk reduction (i.e. an odds ratio of 0.5) in the here proposed study, which would still be of profound clinical relevance. Accordingly, we will need a sample size of 290 patients to detect this treatment effect by a Mantel-Haenszel test with a power of 80% at a two-sided alpha-level of 0.05 (nQuery 7.0). Assuming a dropout rate of about 10% a total of 324 patients should be randomized.

### Compliance/rate of loss to follow up

During the pilot study the reported compliance was markedly high. Of the 111 patients included in the study only 5 discontinued intervention (5%), of whom 2 in the verum group stopped intervention due to potential side effects (dizziness and headache). This high compliance was likely to be related to the high burden derived from the worry about the unborn.

The number of patients calculated for allocation to the PETN trial accounts for a more conservative dropout rate of 10%, due to the particularly good compliance of pregnant women, especially those to be at risk for neonatal complications. For the primary outcome, robustness of the results regarding missing values will also be analyzed.

### Recruitment

**Fig. 1:**
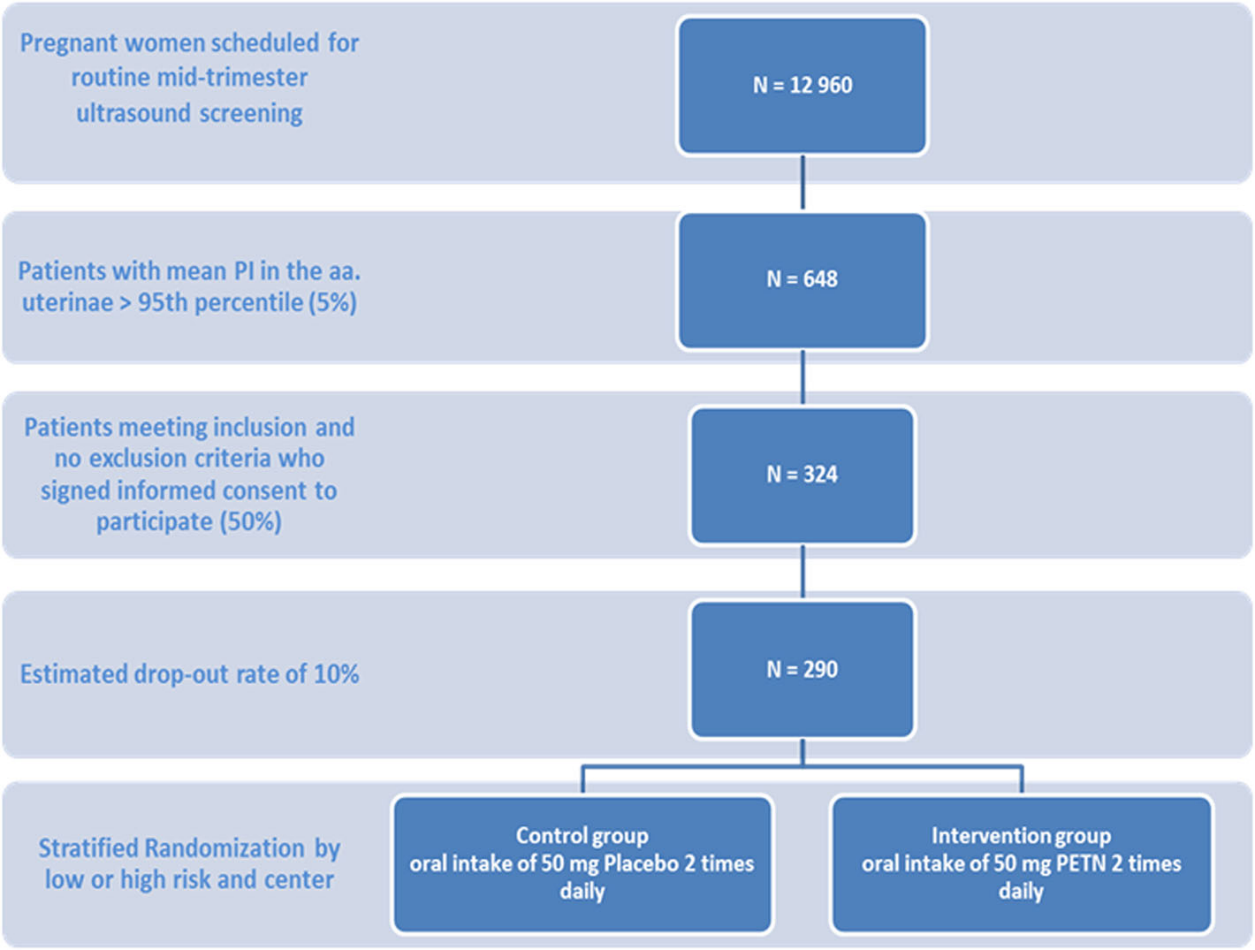
Recruitment plan

Patients eligible are recruited from those pregnant women presenting during prenatal care for routine or risk guided midtrimester ultrasound screening for fetal structural anomalies including the assessment of utero-placental perfusion. 5% are expected to demonstrate impaired utero-placental flow with a mean PI exceeding the 95th centile indicating a risk for IUGR. In our pilot trial an estimate of 50% of the eligible patients were enrolled per year. Accordingly, to recruit 324 patients within 18 months we need to identify 650 women with impaired utero-placental perfusion at mid gestation. Based on the fact, that of all women presenting for mid gestation ultrasound 5% will meet the inclusion criteria (mean PI >95th centile (> 1.6)) a cohort of about 13,000 women will be needed to recruit from. To enlarge the cohort, study centers are asked to cooperate with affiliated outpatient facilities, run by gynecologist holding a DEGUM II or III qualification. Patients recruited in the affiliated facilities will then be sent to the participating study center for verification of eligibility and study inclusion. (Fig. 1).

### Study setting

The study started recruiting in August 2017. The coordinating hospital and trial coordinator is the University Hospital of Jena, PD Dr. Tanja Groten. Participating hospitals and local trial coordinators are University Hospital Schleswig Holstein, PD Dr. Ulrich Pecks; Medical University Hanover, Prof. Dr. Constantin von Kaisenberg; University Hospital Bonn, Mrs. Mateja Condic; Charité Berlin, PD Dr. med. Stefan Verlohren; Vivantes Network of Health GmbH, Clinic Neukoelln, PD Dr. med. Dietmar Schlembach; University Hospital and Polyclinic of Obstetrics and Prenatal Medicine, Dr. med. Gregor Seliger; Obstetric Clinic St. Elisabeth and St. Barbara Hospital Halle, Dr. Sven Seeger; University of Leipzig Medical Center, Division of Obstetrics, Dr. Anne Tauscher; University Hospital Dresden Carl Gustav Carus, Dr. Matej Komar; University Women’s Hospital Tuebingen, Prof. Dr. Karl Oliver Kagan; Ulm University, Clinic of Gynecology and Obstetrics, PD Dr. Ulrike Friebe-Hoffmann; University Hospital of Munich, Dr. med. Christoph Hübener and Municipal Hospital Munich, Dr. Laura de Vries. As of January 2019 206 patients have been enrolled in the study. Enrollment has been expanded to Dezember 2019.

### Experimental and control interventions

Following randomisation, oral intake of PETN or placebo two times daily will be continued until 36^6^ weeks. Maternal and fetal status and condition will be assessed every 4 weeks during the treatment period and at 38^6^ to 40^0^ weeks. (Table 1) Outcome data will be collected after delivery for neonatal (weight, height, APGAR 1′/5′/10′, umbilical artery pH, gestational age at birth, admission to the neonatal intensive care unit, respiratory distress syndrome grade III or IV, occurrence of intraventricular haemorrhage grade III or IV or parenchymal bleeding or periventricular leukomalacia, necrotized enterocolitis, neonatal death) and maternal pregnancy associated morbidity. Additionally, data on child development will be documented on growth, health status and intellectual development at the age of 12 months, by collecting the routine check-up data from the paediatricians. (Table 1).

**Table 1:** Study procedures

Study Period	Recruitment period	Treatment period (12–19 weeks) Study visits fourweekly				Post-Treatment-Follow-up		
Treatment	start at inclusion	oral intake of 50 mg PETN/Placebo 2 times daily				Follow up		Follow up child
Event/Visit	Inclusion visit at 19^0^ to 22^6^ weeks	24^0^ to 24^6^ weeks	28^0^ to 28^6^ weeks	32^0^ to 32^6^ weeks	36^0^ to 36^6^ weeks	38^0^ to 40^0^ weeks	after delivery^h^ (no visit)	Routine check-up at the age of 12 month^i^
Informed Consent	x							
Inclusion/Exclusion Criteria	x							
Randomisation	x							
Ultrasound ^a^	x	x	x	x	x	x		
Maternal status^b^	x	x	x	x	x	x	x (at admission for delivery)	
Haematology/Urinanalysis^c^	x	(x)	x	(x)	x	x	x (if available)	
Maternal condition^d^	x	x	x	x	x	x		
Concomitant medication^e^	x	x	x	x	x	x		
Checking for AEs incl. SAEs		x	x	x	x	x	x	x
Perinatal outcome^f^							x	
Maternal morbidity^g^							x	
Data on child growth and development								x

^a^assessment of fetal growth: head and abdomen circumference, femur length, doppler of aa. aterinae, a. umbilical and a. cerebri media, amniotic fluid index

^b^assessment of maternal status: blood pressure, weight

^c^assessment of haematology and urine: blood count, ASAT, ALAT, LDH, proteinuria and determination of sflt and PlGF amounts and ratio where possible

^d^assessment of maternal condition: head ache, nausea/vomiting, ear or eye symptoms, pain in the upper abdomen, oedema, others

^e^concomitant medication: name, dosage, frequency, duration, reason of intake

^f^assessment of perinatal outcome: weight, height, growth centile, APGAR 1`/5`/10`, pH of umbilical artery, exact gestational age, admission to neonatal intensive care unit, respiratory distress syndrome, occurrence of intraventricular haemorrhage, necrotized enterocolitis, neonatal death

^g^assessment of maternal morbidity: e. g. Preeclampsia, HELLP syndrome, abruption placentae

^h^no visit required, post-delivery data will be collected as copies of health reports, discharge or pregnancy reports

^i^Follow up of the children will be collected as copies of the mandatory standard care visit at the pediatrician at the age of 1 year

### Assessment of safety

Monitoring of selected safety data (maternal serum and clinical parameters, fetal growth control and control of utero-placental and fetal perfusion) is scheduled every 4 weeks. Monitoring of Adverse Events (AEs) and Serious Adverse Events (SAEs) is established. A data safety monitoring board is constituted to supervise safety events and outcome data of the neonates born during the ongoing study. The data safety monitoring board has met on a regular basis; three times so far, did not find any peculiarities and had no recommendations on protocol changes.

### Statistical analysis

Baseline characteristics of patients will be compared by descriptive statistics to assess the success of randomization. The primary endpoint (binary variable) will be presented by group-specific incidences. The confirmatory test of the treatment effect stratified for risk-groups will be performed by the Mantel-Haenszel test. Mantel-Haenszel estimates of relative risks and their 95% confidence intervals will be calculated. Primarily, data will be analyzed according to the intent-to-treat (ITT) principle. The ITT-population will include all randomized patients with valid primary outcome data who have received at least one dose of study medication (full analysis data set). The drop-out rate is expected to be 10%. Missing values of the primary endpoint are multiply imputed to confirm the robustness of the main results (sensitivity analysis). All secondary endpoints (binary variables) will be analysed by Mantel-Haenszel methods as described above. In a sensitivity analysis generalized mixed linear models with treatment and risk indicator as fixed, and centre as random factor, will be fitted to adjust for variability between study centres. Explorative analyses of endpoints will be additionally performed for subgroups according to time of delivery and maternal age. AEs/SAEs will be listed. Incidence of events and descriptive statistics (quartiles, mean, standard deviation) of laboratory safety data will be displayed in treatment groups. No adjustment for endpoint multiplicity is planned. All tests will be done at a comparison-wise significance level of 0.05.

## Discussion

FGR is the most important determinant of perinatal outcome in pregnancies with impaired utero-placental perfusion, at worst resulting in perinatal death. The relevance of reducing the occurrence, rate and extent of FGR is obvious in light of the associated outcomes. FGR neonates have an incredible risk to develop neonatal complications and lifelong health effects [2]. A reduction of this burden and the additional decrease in the rates of prematurity and perinatal death will be of tremendous benefit for the affected women. Furthermore, reduction of FGR and prematurity might additionally decrease the long-term consequences like development of diabetes and obesity in the affected children, thereby also contributing to relieve the general health system. The chosen composite endpoint represents the most severe outcome possible in growth restricted pregnancies and is justified by being the endpoint of our pilot study.

The management of FGR so far is driven by an approach to delay delivery, since prematurity is the most important determinant for perinatal outcome and lifelong health in growth restricted newborns. Nevertheless, in cases where intrauterine hypoxia is rising and intrauterine asphyxia and death is threatening, preterm delivery is required to save the baby’s life. Thus, prematurity is often associated with FGR, thereby deteriorating the infants long-term prognosis. The clinical parameters accepted to determine long-term clinical outcome of preterm children are the occurrence and severity of intracerebral bleeding, severe bowel affection (necrotized enterocolitis) and the long-time lung function which is determined by the fetal respiratory distress syndrome [17–19]. Therefore, preterm birth and very early preterm delivery, as well as perinatal outcome data was chosen as secondary endpoints.

Eligible patients for the proposed trial are pregnant women recognized to be at risk of adverse pregnancy outcome associated with FGR at mid gestation by impaired uterine perfusion. In the absence of a general screening for placentation failure by Doppler measurement at mid gestation in all pregnant women, those recognized with impaired placental perfusion are more likely to present with additional risk factors and do not represent the general population. However, since the effectiveness of PETN to substantially improve pregnancy outcome has been especially profound in high risk women, these women are the one targeted by this study. Additionally, these women, often with a history of severe adverse pregnancy outcome, are particularly in need of treatment options. Since there are still low risk women to be included in the study, reported evidence for these women, will still be representative for a general population of pregnant women.

For obstetricians worldwide the management of FGR is still lacking therapeutic options. During the past decade therapeutic agents with possible perfusion optimizing potential have been evaluated in clinical trials.

Antiplatelet agents, like aspirin have been extensively investigated for prevention of preeclampsia and also FGR. A meta-analysis summarizing individual patient data of more than 32.000 women in 31 randomized trials showed a reduction in the relative risk of birth before 34 weeks’ gestation and of preeclampsia, but not of perinatal death or FGR [20]. Recently data from the ASPRE trial where the effect of 150 mg aspirin to prevent preeclampsia in high risk women was investigated also showed a reduction of FGR cases in women developing preeclampsia [21, 22] when started before 16 weeks of gestation [23].

Sildenafil citrate, a phosphodiesterase-5 (PDE-5) inhibitor preventing the degradation of the second messenger cyclic guanosine 3 ′,5 ′ -monophosphate by the enzyme PDE-5, is a potent vasodilator and has been proposed for the treatment of FGR pregnancies [24–26]. First results of an international randomized controlled trial, including women with diagnosis of FGR beyond 26 weeks of gestation (STRIDER trial) has so far failed to show therapeutic effects [27].

Lees and co-authors demonstrated the clinical effect of NO-donors in pregnant women at risk before [11]. In a randomized placebo-controlled blinded trial he demonstrated an increased likelihood for complication free pregnancy upon administration of glycerol trinitrate (GTN). Due to the small sample size the study, however, failed to demonstrate significant effects for the primary reduction in FGR, preterm delivery and PE. Furthermore, the development of nitrate tolerance was observed within a few days, could additionally limit the clinical effect [28].

Recently, the group of Valensise showed an improvement of maternal hemodynamics in complicated pregnancies when NO-donors were added to the initiated hypertensive therapy. The authors conclude that, as a consequence of changes in maternal hemodynamics, utero-placental perfusion is ameliorated and thus pregnancy outcome improved [29]. Complementary, it was shown by our group before, that NO-donors improve placental perfusion without affecting fetal circulation [30].

Valensise and colleagues studied the effect of the addition of NO-donors to antihypertensive therapy in pregnancy, showing significant enhancement of maternal hemodynamics and plasma volume expansion, faciliating prolongation of pregnancy. By enhancing materno-placental perfusion NO donors reduce pregnancy complications associated with impaired perfusion [29].

PETN, however, has notably peculiar features which differentiate it from all other organic nitrates, such as nitroglycerin, isosorbide-5-mononitrate, and isosorbide dinitrate. Upon treatment with PETN no develop a nitrate tolerance can be observed, probably due to its specific impact on intracellular signalling pathways [28, 31]. PETN increases the expression of the antioxidant genes HO-1 and ferritin heavychain (FeHc) in human endothelial cells. These increase in HO-1 and FeHc expression potentially impact on mitochondrial ROS production and the inhibition of mitochondrial aldehyde dehydrogenase activity and thus the avoidance of tolerance developement [28]. So far it remains speculative, whether reduced ROS production and enhanced HO-1 expression might also serve as a mechanism provoking improved endothelial cell function and thus vascular health.

To prove the effects of PETN on adverse pregnancy outcome, particularly the significant decrease in the incidence of FGR and preterm birth, as well as to confirm the the efficacy and safety of PETN, a randomized, double-blinded, placebo-controlled, multi-center trial was initiated.

## Data Availability

The datasets used and/or analysed during the current study are available from the corresponding author on reasonable request.

## Abbreviations

AE/SAE: Adverse Events and Serious Adverse Events
FeHc: Ferritin heavychain
FGR: Fetal growth restriction
GTN: Glycerol trinitrate
HO-1: Heme oxygenase-1
ITT: Intent-to-treat
LDA: Low-dose aspirin
NO: Nitric oxide
PDE-5: a phosphodiesterase-5
PETN: Pentaerithrityl tetranitrate
PI: Pulsatility Index
